# Whole-body magnetic resonance imaging in children – how and why? A systematic review

**DOI:** 10.1007/s00247-020-04735-9

**Published:** 2020-06-25

**Authors:** Pia Zadig, Elisabeth von Brandis, Regina Küfner Lein, Karen Rosendahl, Derk Avenarius, Lil-Sofie Ording Müller

**Affiliations:** 1 Department of Radiology, University Hospital of North Norway, Sykehusvegen 38, 9019 Tromsø, Norway; 2 University of Tromsø – The Arctic University of Norway, Tromso, Norway; 3 Department of Radiology, Oslo University Hospital, Oslo, Norway; 4 Medical Library, University of Bergen, Bergen, Norway; 5 Department of Radiology and Intervention, Unit for Paediatric Radiology, Oslo University Hospital, Oslo, Norway

**Keywords:** Adolescents, Children, Indications, Magnetic resonance imaging, Systematic review, Technique, Whole-body magnetic resonance imaging

## Abstract

Whole-body magnetic resonance imaging (MRI) is increasingly being used for a number of indications. Our aim was to review and describe indications and scan protocols for diagnostic value of whole-body MRI for multifocal disease in children and adolescents, we conducted a systematic search in Medline, Embase and Cochrane for all published papers until November 2018. Relevant subject headings and free text words were used for the following concepts: 1) whole-body, 2) magnetic resonance imaging and 3) child and/or adolescent. Included were papers in English with a relevant study design that reported on the use and/or findings from whole-body MRI examinations in children and adolescents. This review includes 54 of 1,609 papers identified from literature searches. Chronic nonbacterial osteomyelitis, lymphoma and metastasis were the most frequent indications for performing a whole-body MRI. The typical protocol included a coronal STIR (short tau inversion recovery) sequence with or without a coronal T1-weighted sequence. Numerous studies lacked sufficient data for calculating images resolution and only a few studies reported the acquired voxel volume, making it impossible for others to reproduce the protocol/images. Only a minority of the included papers assessed reliability tests and none of the studies documented whether the use of whole-body MRI affected mortality and/or morbidity. Our systematic review confirms significant variability of technique and the lack of proven validity of MRI findings. The information could potentially be used to boost attempts towards standardization of technique, reporting and guidelines development.

**Electronic supplementary material:**

The online version of this article (10.1007/s00247-020-04735-9) contains supplementary material, which is available to authorized users.

## Introduction

Whole-body MRI is a relatively new assessment tool that allows for imaging of the entire body in one scan, with the potential to provide both anatomical and functional information. Without the use of ionizing radiation, it enables the depiction of disease at an early stage, sometimes even before the onset of clinical symptoms. This ability to detect clinical silent lesions is often highlighted as an important feature of whole-body MRI [1–6]. The value of whole-body MRI as an advanced method to evaluate disease in adults has been investigated by several authors [7–10]; however, the results drawn from these studies are not directly applicable to children. For example, when searching for bone diseases, previous research has shown that MRI findings in the wrist and feet believed to represent pathology in the mature skeleton merely represent normal, growth-related changes in children [11–17]. Knowledge of normal signal changes in paediatric bone marrow is crucial for the interpretation of whole-body MRI to avoid false-positive lesions and thereby overstaging of disease, particularly when searching for clinically silent lesions. To our knowledge, no studies addressing normal bone marrow signal changes on paediatric whole-body MRI have been published. Moreover, studies addressing the precision, accuracy and clinical validity of whole-body MRI in children are sparse [18].

Despite these shortcomings, whole-body MRI is increasingly being used in children with suspected chronic nonbacterial osteomyelitis, Langerhans cell histiocytosis and malignancies, amongst others [19, 20]. Even though the whole-body MRI protocol should, to some extent, be tailored to the specific indication, a standard whole-body MRI protocol can be used for most practical purposes and indications. At least minimum requirements for parameters (e.g., slice thickness) can be defined. Protocols seem to vary significantly across institutions, with respect to sequences, planes and imaging parameters and there is no uniform or standard protocol for whole-body MRI imaging [19–21]. Further, the nomenclature regarding anatomical coverage in these examinations is inconsistent [20, 22, 23] and a unifying interpretation and reporting system is lacking.

Several reviews on paediatric whole-body MRI address potential indications and sequences used [19–21, 24–26], however, hitherto, no systematic review has been published. We therefore aimed to provide a systematic review of the literature to describe common indications and protocols used to assess multifocal disease, and to examine the diagnostic value of whole-body MRI in children and adolescents.

## Materials and methods

### Information sources and search

We conducted literature searches in Medline, Embase and Cochrane for published papers about whole-body MRI of children and adolescents until November 2, 2018. Relevant subject headings and free text words were used for the following concepts: 1) whole-body, 2) magnetic resonance imaging and 3) child and/or adolescent. The detailed search strategies are given in Online Supplementary Material 1.

### Study selection

Title and abstracts of all identified papers were read by two radiologists (P.Z., a radiology resident, and E.v.B., with 10 years of experience) and checked for eligibility. The selected papers from this phase plus two papers identified through other sources were read in full text by the same radiologists and scored as being eligible for inclusion, not eligible or uncertain. In cases of disagreement or uncertainty, consensus was obtained by discussion.

### Criteria for inclusion and exclusion

Studies reporting on the use and/or findings from whole-body MRI examinations to assess multifocal disease in children and adolescents up to 19 years old were included. For studies including both children/adolescents and adults, they were included if data on at least 10 children/adolescents could be identifiable separately. Studies written in English and with the following study designs were included; cross-sectional, case control, cohorts or randomized controlled trials.

We excluded postmortem studies and whole-body MRI used for body fat composition, body stature or muscle mass quantification. Clinical studies using whole-body MRI to evaluate fat infiltration of muscle, e.g. in neuromuscular diseases, and studies using whole-body MRI angiography or whole-body integrated MRI/positron emission tomography (PET) were also excluded.

### Data and variables

Information on study and patient characteristics, including methodology and indications for performing whole-body MRI and the imaging protocol with technical parameters, were extracted from the included studies. The following technical parameters were registered: field strength, sequences used, scan plane, slice thickness, repetition time (TR) and echo time (TE) for T1-weighted (T1-W), fluid sensitive sequences (T2-weighted/short tau inversion recovery [STIR]) and diffusion-weighted imaging (DWI) and inversion time for STIR sequences and b-values for DWI. If possible, acquired voxel volumes were extracted, either directly or calculated. The voxel size was calculated from the field of view (FOV) and the matrix, using a scan percentage of 100%. We sought to extract the acquired voxel volumes, as a measure for others to repeat the studies. When the FOV was given as two different values, the highest number was used to calculate the voxel size in the frequency direction. When the FOV was given as a range, the mean value was used. We registered the use of contrast, sedation and anatomical coverage. For T2-weighted (T2-W), STIR and DWI sequences, we registered whether motion artefact reduction methods were used. We also assessed the variation in total scan time and the use of audio or video during scanning. All included studies were checked for information about intra- and interobserver agreement and if imaging findings were validated. In addition, we registered papers that mentioned clinically silent lesions (related to expected pathology) and incidental findings (unrelated to known or expected pathology), and whether the possibility of these findings being normal variants was discussed. Also registered was whether the use of whole-body MRI affected mortality and/or morbidity. We also investigated if some studies attempted to establish a standard reporting or scoring system for whole-body MRI. Finally, we registered if a radiologist was amongst the authors and in which journals the papers were published.

If some of the information we sought was unclear, we tried to find the missing information from images or figure legends. When available, supplemental material was read, and additional information was extracted. Information about the scan parameters and resolution was checked against the paper text. The values were not registered if the information was contradictory.

### Statistics

Data were exported into SPSS (Statistical Package version 26; IBM Corp., Armonk, NY) and cleaned. Frequencies were given as numbers, with appropriate measures such as percent, proportions or ratios.

## Results

**Fig. 1:**
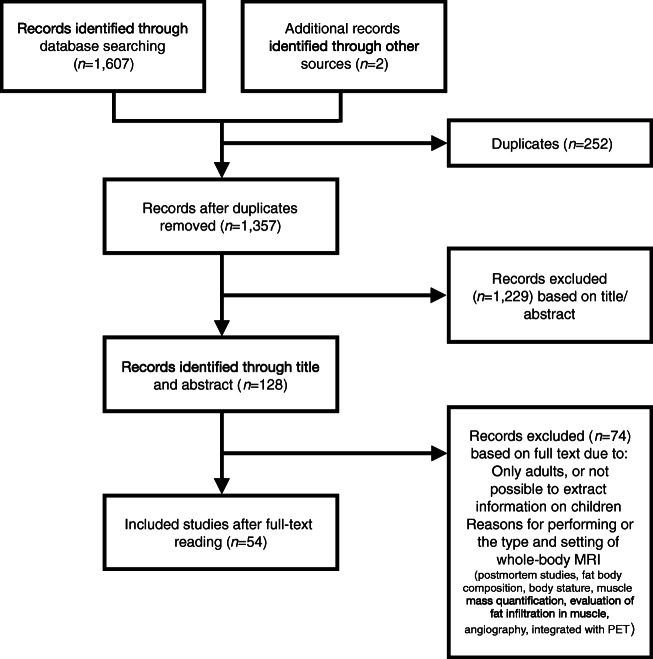
Flow diagram for study exclusion and inclusion

**Fig. 2:**
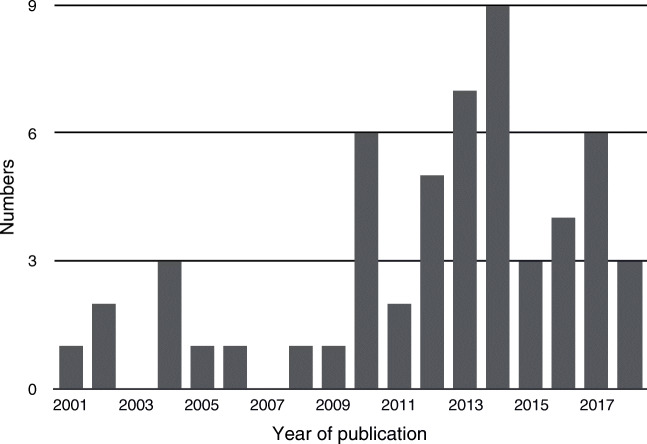
Included papers by year of publication

After removing duplicates, 1,357 publications were screened by reading titles and abstracts. The 128 judged possibly eligible were read in full text. Of the 54 papers included [1–3, 5, 6, 27–75] (Fig. 1), 78% were published during 2010–18 (Fig. 2). The majority of included studies (98.2%) focused mainly of children and adolescents (up to 18 years old). Forty papers (74.1%) were exclusively about children and adolescents, and in 13 papers (24.1%) the focus was on both children/adolescents and adults, but the majority of patients were still younger than 19 years old. The majority of participants were adults in only one of the included papers [42].

### Study and patient characteristics

Thirty out of the 54 studies (55.6%) had a retrospective design whilst 24 (44.4%) were prospective. None of the included studies was a randomised clinical trial. The number of individuals who underwent one or more whole-body MRI in each included paper varied from 7 to 82 (mean: 26, standard deviation [SD] 17). The mean age was given in 38 papers, and it ranged from 0 to 19 years; in 24 papers (60.5%), it ranged from 10 to 15 years. The studies included 1,829 whole-body MRI examinations (mean: 33, SD 22). Fifteen studies (27.8%) reported on protocols adjusted to child size, indication, vendor or field strength, and these protocols were examined separately.

### Indications

Based on the included studies, we found chronic nonbacterial osteomyelitis (15 papers), lymphoma (8 papers) and cancer staging (8 papers) to be the most frequent indications for performing a whole-body MRI. All examinations had the purpose of diagnosing and/or evaluating the extent of disease. In six papers, the indications for the study participants to undergo whole-body MRI differed, and were consequently registered as multiple indications (Table 1). One paper, for example, included cases of suspected child abuse, chronic nonbacterial osteomyelitis, lymphoma, osteosarcoma, neuroblastoma and Langerhans cell histiocytosis [59].

**Table 1:** Indications for performing whole-body MRI in studies included in our review

Indication	Number of papers
Chronic nonbacterial osteomyelitis	15
Lymphoma	8
Metastasis	8
Cancer predisposition syndrome disease	4
Juvenile dermatomyositis	2
Osteonecrosis	3
Neurofibromatosis	3
Langerhans cell histiocytosis	1
Nonaccidental injury	1
Healthy term infants	1
Sickle cell disease	1
Acute myeloid leukemia	1
Multiple indications in the same paper (i.e. cystic angiomatosis, Langerhans cell histiocytosis, lymphoma, neuroblastoma, rhabdomyosarcoma, Ewing tumor, chronic nonbacterial osteomyelitis, suspected child abuse, etc.)	6

### Field strength

Of the 54 studies, 34 (63%) used a 1.5-tesla (T) system only, while 6 (11%) used 3 T only. Three of the 54 studies (5.5%) used both 1.5 T and 3 T. Eleven studies did not give any information on field strength.

### Protocol

The typical whole-body MRI protocol included STIR sequences only (20 papers) [29–32, 38, 39, 41–44, 51, 56, 58, 61, 63, 66–68, 74, 75] or STIR and T1-W sequences (17 papers) [2, 3, 5, 27, 28, 35, 37, 40, 45, 46, 49, 50, 55, 57, 60, 62, 71]. Five studies used a protocol including STIR, T1-W and DWI [36, 54, 59, 64], and four studies used only STIR and DWI [52, 53, 69, 70]. In one study, whole-body MRI was performed on equipment from different vendors with different protocols; the use of STIR was mentioned, but no information about the use of T1-W or DWI was given [73]. In two studies, the term STIR only appeared in a legend, and no information was given on whether the study included T1-W or DWI [1, 72]. In two of the studies, information on sequences was unavailable [6, 65].

### Radiofrequency coils

In 9 studies, the body coil was used as the receiver coil. In 18 studies, dedicated surface coils were used. Twenty-seven papers did not give information on coil type.

### Technical settings

Forty-nine out of 54 studies (90.7%) used fluid-sensitive sequences, of which 48 used STIR. One of the studies used a single-shot T2 sequence and three papers did not report on whether a fluid-sensitive sequence had been used. Thirty-nine of the 48 studies using STIR performed the scan in the coronal plane. One study examined protocols from different institutions, and T2 Dixon/T2 fat saturated was mentioned as an alternative to STIR, although this was used in only a few cases [73]. FOV in the feet-head direction varied from 200 to 500 mm per sequence. For papers providing detailed information on the technical settings, TR, TE, STIR inversion time and slice thickness varied substantially (Table 2). Two of the 48 studies (4.2%) using STIR sequences gave the acquired voxel volume, and for 14 studies (29.2%), this parameter could be calculated from the FOV and the matrix (as described in the Materials and methods section).

**Table 2:** Technical parameters at 1.5 T and 3 T used for whole-body MR performed in children and adolescents for various indications, based on a systematic review of the literature up to November 2018

	1.5 T Range (mean) (SD) (number of papers)			3 T Range (mean) (SD) (number of papers)		
**Parameters**	T2-weighted/STIR^a^	T1-weighted fast spin echo	Diffusion-weighted	T2-weighted/STIR^a^	T1-weighted fast spin echo	Diffusion-weighted
**Repetition time (ms)**	800–8,500 (3,897.3) (2,014.5) (29)	85–1,610 (608.5) (394.8) (10)	4,500–8,612 (6,892.1) (1,602.3) (6)	2000–9,126 (4664) (2,033.2) (6)	480–820 (694.3) (186.5) (2)	6,100–7,200 (6,566.7) (568.6) (2)
**Echo time (ms)**	17–284 (65.1) (52.9) (29)	7–18 (12) (2.9) (10)	62–78 (72.6) (6.2) (6)	30–326 (87.9) (98.2) (6)	9–12 (10.3) (1.5) (2)	55–62 (58.5) (5) (2)
**STIR inversion time (ms)**	64–180 (148.4) (21.2) (27)	–	–	160–230 (205) (5)	–	–
**Slice thickness (mm)**	1.3–10 (5.7) (1.8) (29)	4–9.5 (5.9) (1.8) (10)	4–6 (4.3) (0.8) (6)	3–8 (5.2) (1.6) (6)	4–5.5 (4.8) (0.8) (2)	4–4.5 (4.3) (0.4) (2)
**Acquired voxel volume (mm**^**3**^**)**	2.7–45 (18.4) (13.2) (14)	7.5–45 (21.8) (14.6) (6)	19.2–82.8 (57.6) (23.3) (3)	7.8–11.3 (9.1) (1.9) (3)	6.1^b^	15^b^

^a^ Combined data from all fluid-sensitive sequences

^b^ Number of papers was one, thus mean and SD could not be calculated

*SD* standard deviation, *STIR* short tau inversion recovery

A T1-W sequence was included in 24 studies (44.4%), of which 15 (62.5%) were performed in the coronal plane. FOV in the feet-head direction varied from 265 to 500 mm. Fourteen of the 24 studies (58.3%) used fast spin-echo sequences and the TR, TE and slice thickness varied greatly amongst these studies (Table 2). In two studies, T1 was performed as a gradient echo sequence. One of the studies (7.1%) using fast spin-echo sequences reported the acquired voxel volume; for 6 studies (42.9%), it was calculated.

Ten of the 54 studies (18.5%), all of which were published after 2012, included a DWI sequence. For 8 of the 10 studies, DWI was performed in the axial plane. We could not assess information about the scan plane in the two remaining papers. In 1 of the 10 papers, the acquired voxel volume was given and in 3 it could be calculated from the parameters (Table 2). All 10 studies used more than 1 b-value and the highest b-value was 1,000.

### Contrast enhancement

Five of the included studies used contrast-enhanced sequences as part of the whole-body MRI protocol [5, 37, 40, 47, 62]. A sixth study used contrast, but it was unclear whether this was part of the whole-body protocol [71]. Five of the six used gadolinium and one study used iron oxide nanoparticles [47].

### Sedation and audio/video

In 16 of the 54 studies (29.6%), sedation was given. Eleven studies did not use sedation and in 27 papers (50%) this information was not provided. None of the included papers reported the use of audio or video during the whole-body MRI examination.

### Artefact reduction techniques

Artefact reduction techniques were used in 1 of the 10 protocols with DWI (10%) and in 11 protocols with fluid sensitive sequences (22.4%). The different artefact reduction techniques were breath-hold and respiratory triggering, electrocardiographic gating and buscopan as an antispasmodic agent.

### Scan time

Scan time was defined differently in the studies and varied between sequence time, total MR machine time and time spent in the department. It was not possible to extract sufficient information about the length of the individual sequences used.

### Body coverage

The whole body from head to toe was included in 32 studies (59.3%). In nine of the papers, no information on coverage was given. In the remaining studies (24.1%), the body coverage varied from head to pelvis or head to thigh.

### Reliability and validation of findings

Ten of the studies (18.5%) performed repeatability tests, of which 2 studies assessed inter- and intra-observer agreement [32, 56] and 8 examined for interobserver agreement only [5, 38, 47, 52, 54, 59, 61, 67]. Their results varied depending on what was assessed from the whole-body MRI. In the two studies assessing inter- and intra-observer reliability, the intra-observer agreement was higher than the agreement between the readers. Two studies looked at interobserver agreement for both nodal and extranodal sites, and scoring of the nodal sites had a better agreement than the extranodal, including bone marrow lesions [52, 54]. Thirty-nine studies (72.2%) did not perform repeatability tests and in 5 of the 54 included studies there was no information on inter- and intra-observer agreement. Thirty-two studies (59.3%) compared whole-body MRI findings with an established reference standard, i.e. histology/biopsy (reference standard) and/or observation including biopsy and follow-up imaging. Eighteen studies (33.3%) did not validate their findings. In four studies, no information on validation was given. None of the studies performed biopsy of all lesions that were scored as pathology on whole-body MRI.

### Interpretation/reporting system

Several of the papers included in this review described the imaging criteria they used to define pathology. However only one paper, from 2017 by Arnoldi et al. [28] proposed a standardized reporting system for whole-body MRI for patients with chronic nonbacterial osteomyelitis.

### Incidental findings, clinically silent lesions and normal variants

Six papers reported on incidental MR findings [5, 27, 29, 33, 36, 64]. In the study from Anupindi et al. [27], 55 incidental findings were detected in 23 of 24 patients (96%). None of the findings was of significant clinical impact requiring imaging follow-up or treatment. The remaining five papers did not provide any details on incidental findings.

In 18 of the papers (33.3%), the authors concluded that whole-body MRI is a sensitive tool for detecting clinically silent lesions [1–3, 5, 6, 28, 32, 35, 37, 40, 43, 46, 56, 60, 62, 72, 73, 75], of which only 3 papers discussed the possibility of these findings being due to normal growth related changes [35, 37, 75]. In total, 12 of 54 papers (22.2%) suggested bone bruise and/or highly cellular bone marrow as a cause of high signal on T2-W sequences [34, 35, 37, 45, 50, 51, 53, 57–59, 66, 75]. One paper mentioned in a figure legend that diffuse high signal of the bone marrow at DWI is a normal finding in the spine and pelvis in children [54].

### Effect on long-term disease course

None of the included studies addressed whether whole-body MRI affected long-term disease course (mortality/morbidity).

### Authors and journals

Fifty of the 54 papers had a radiologist amongst the authors, in 30 of these (55.6%) as a first author. Sixteen papers (32.7%) contained sufficient technical parameters for the reader to reproduce the T2-W/STIR resolution, whereof 14 (87.5%) had a radiologist as the first author and were published in radiology journals. In total, 30 of the included papers (55.6%) were published in radiology journals.

## Discussion

Our results show that whole-body MRI is commonly used in many institutions, particularly to assess chronic nonbacterial osteomyelitis, lymphoma and malignancy/metastasis. However, the body area covered, the sequences used and the technical details vary significantly. More importantly, only a few studies address the validity of MRI findings.

We found that the term “whole-body MRI” is used interchangeably for examinations covering from two-thirds to the whole body. For instance, whole-body MRI performed for staging of malignant disease sometimes covers skull-base to mid-thigh only [42, 52, 54, 67–69], similar to what is often being used for PET-CT. According to some authors, important additional clinical information, like therapy-related osteonecrosis of the knees/ankles [55] or distant metastases in the extremities [73] could be missed due to this limited FOV, hence a clear definition of what should be covered in the scan must be defined when recommending whole-body MRI for specific diseases. To standardize the nomenclature, we agree with Greer et al. [25] in defining whole-body MRI as a “multiregional contiguous imaging of the entire body” and arguing that the term “whole-body MRI” should be reserved for “head-to-feet or vertex-to-heel imaging unless otherwise specified” [23]. Moreover, Greer et al. [23] states that “where smaller FOV is sufficient, whole-body MRI can be truncated and should be annotated accordingly.”

The current literature does not allow us to give any preferences on field strength for whole-body MR imaging in children, i.e. whether a 3-T system performs better than a 1.5-T system. This issue was addressed in one study only including 21 children with different tumors, examined on both 1.5-T and 3-T systems [61]. They found substantial agreement between two observers, but a better image quality on the 1.5 T, and also fewer artefacts. They concluded that this difference was unlikely to be significant as the image quality of the 3-T protocol was scored high enough. It is, however, difficult to compare field strength as such because the resultant image also depends on the machine hardware, software, coil and the protocol used.

Most protocols included coronal STIR and T1-W sequences, but the technical settings varied considerably across institutions. The current use of STIR sequences was not unexpected, as this is a particularly robust, widely used imaging method that may improve lesion detection. However, recent technical improvements have resulted in methods with better signal to noise ratios, such as T2-W with spectral fat saturation and T2-W Dixon, allowing for higher image resolution. In the present review, only one study mentioned T2-W Dixon, however, with no elaboration on a potentially added value [73]. The Dixon technique provides images with and without fat suppression and fat only images, which may replace the T1-W fast spin echo (FSE) images for bone marrow pathology [76]. This may reduce examination time, which is particularly beneficial in children.

One might argue that varying technical settings for the different pulse sequences, such as TR, TE and image resolution, is inevitable due to different scanners, indications, coils, body sizes, etc., and that especially the TR of T2-W/STIR sequences often is irrelevant. However, we found that TR and TE times of T2-W/STIR sequences varied significantly (Table 2), and to such an extent that it might influence image quality and, more importantly, image analysis. A short TR of 800 ms in a T2-W sequence can result in low signal-to-noise ratio and additional T1 weighting, while a short TE of 17 ms in a T2-W sequence gives less T2 and more proton weighting. The upper range of TE for the T2-W/STIR sequences was higher than expected and could result in low signal-to-noise ratio. This would reduce image resolution and, consequently, decrease lesion detection. Further, variation in STIR inversion time influences the degree of fat suppression, whilst high TR times in T1 sequences minimize the T1 weighting. We believe that defining a size- or weight-based standard protocol for whole-body MRI within institutions works for most practical purposes and indications. We also believe that defining standard and/or minimum requirements for technical settings, e.g., maximum slice thickness (3–4 mm depending on patient size or weight) across institutions to ensure sufficient diagnostic quality and to perform comparative studies, is warranted.

The use of DWI as part of the whole-body MRI has been addressed in several studies on adults, however, only 10 of the current studies included a DWI sequence. Five of the studies assessed a potential added value of DWI in addition to anatomical sequences. Three of these studies concluded that, in contrast to what has been shown for adults, there was no significant benefit in diagnostic accuracy from adding DWI sequences [52, 54, 59]. According to the authors, this could be related to the small number of patients in the current studies or to child-specific issues such as smaller body-size or challenges in interpretation of bone marrow signal in children due to inhomogeneous DWI signal. Two papers concluded that DWI could add useful information [53, 69]. The study by Leclair et al. [53] showed a substantial elevation of the apparent diffusion coefficient (ADC) values in inflammatory bone lesions due to chronic recurrent osteomyelitis (chronic nonbacterial osteomyelitis). Hence, the authors highlighted DWI as a promising technique that may help to distinguish benign inflammatory processes from malignant lesions. However, they only assessed chronic nonbacterial osteomyelitis lesions. Furthermore, they also mentioned the limitations of DWI in children due to high bone marrow signals. Punwani et al. [69] investigated the added value of DWI to PET to predict local treatment response in children and adolescents with Hodgkin lymphoma. Pretreatment ADC values in nodal masses with adequate response were significantly lower than in sites with inadequate response. Based on their results, the authors suggested that DWI should be incorporated in integrated PET/MRI protocols [69].

This review revealed inconsistent, and often suboptimal, reporting of MR technical settings. Most often, a combination of FOV, imaging matrix and slice thickness was provided. For some protocols, ranges of FOV and slice thickness were given, as these parameters were adjusted, according to the region scanned. Numerous studies lacked sufficient data for calculating image resolution, making it impossible for the reader to reproduce the protocol/images. In our opinion, details to assess the acquired voxel size should be provided routinely when reporting on whole-body MRI. The acquired voxel size was reported in only a few papers, while from some papers the reader could deduce the acquired voxel size based on given parameters, and by an assumption of the scan percentage. We used a scan percentage of 100% to calculate the in-plane resolution, although it is more common to use a scan percentage of around 80% since this reduces scan time. However, this means that our calculated voxel sizes can be slightly smaller than what was used in the protocol, and underestimation of the resolution was thus avoided.

The voxel size used for the T2-W/STIR sequences varied by a factor of 20. None of the studies, however, compared the effect of different voxel sizes on image quality, or, more importantly, on the imaging findings. One study, however, proposed a high-resolution 3-D STIR scan as the sole sequence, and compared this to a protocol with both 3-D STIR, T1 and DWI [59] concluding that the protocols were equally good at detecting pathology. The technical improvements of MR equipment, software and coils make it increasingly feasible to image with higher resolution, although we found no significant trend when comparing voxel size and year of publication. It is possible that some sites would rather perform more sequences, acquisitions in more imaging planes, or shorten total scan time, rather than choosing a higher resolution scan. We were not able to extract enough information on actual scan times.

Detailed information on the coils used was sparse. Some authors used a body coil. Although comfortable for the child, this coil gives a low signal-to-noise ratio, thus hindering high-resolution imaging. Dedicated coils, such as phased array coils allowing for high resolution imaging, were used in several of the studies. The variety of coils used may, in part, explain the wide range of image resolution found for all types of sequences.

Only a few studies reported on child-specific issues such as the use of sedation and/or motion artefact reducing methods, be it technical or practical such as watching films or listening to music. For papers by clinicians in non-radiologic journals this is understandable, however, many of the radiology-driven studies also lacked this important information enabling others to reproduce the examination.

In the adult literature, several studies report on the frequency of incidental whole-body MRI findings [10, 77, 78], but in children this information is sparse. In the current review, only one study addressed this issue, reporting a prevalence of 96% [27]. In general, there is no published consensus on how to manage incidental findings on whole-body MRI [10]. Moreover, due to the lack of normal reference standards for whole-body MRI in children, it may be difficult to define the findings that have “potential health or reproductive importance” [79].

Amongst the papers included in the current review, several concluded that the sensitivity of whole-body MRI for detecting bone-marrow lesions is superior to that of conventional imaging methods [2, 34, 38, 62, 70]. Further, several authors emphasized the method’s ability to detect clinically silent lesions, thus without discussing the risk of false-positive findings due to growth-related signal variation. Amongst these were two papers describing any bone marrow hyperintensities as either related to disease [41] or therapy [43] without giving their definition of pathology.

The lack of standardized interpretation of whole-body MRI findings may lead to misinterpretation and subsequent consequences for diagnosis and treatment. This is illustrated by the study of Zibrowska-Bech et al. [3], who retrospectively evaluated 31 children diagnosed with chronic nonbacterial osteomyelitis over 10 years [3]. One of the children in the study was erroneously treated with chemotherapy for 2 weeks under the suspicion of having osteosarcoma, before the diagnosis was changed to chronic nonbacterial osteomyelitis. Another child had initially been misdiagnosed with Langerhans cell histiocytosis and was treated with vinblastine and prednisolone for 6 months before a chronic nonbacterial osteomyelitis diagnosis. In both cases, there was a substantial diagnostic delay, ranging from 8.4 months in the first case to 37 months in the second case.

Only a few studies performed reliability tests, but both papers reporting on inter- and intra-observer agreement showed a substantially higher score for intra-observer agreement, in part reflecting the lack of definitions for MRI pathology. Opposite, the interobserver agreement for evaluating nodal versus extranodal pathology, clearly defined by international standards, was good [52, 54].

Histology was used in 17 studies to validate the accuracy of the whole-body MRI findings. For obvious reasons, not all findings on MRI could be biopsied, hence some of the lesions detected on whole-body MRI could still be normal findings or unrelated to the underlying disease.

There are several limitations to this review. Important information from non-English publications or from studies by the clinical site without indexing “whole-body MRI” may have been missed. Another potential bias is the exclusion of combined adult-paediatric studies with insufficient information on the included children/adolescents. It was not possible to extract information about average scan time due to a lack in data. Lastly, studies listing multiple indications were registered as “multiple,” thus decreasing specificity.

## Conclusion

Whole-body MRI is being used in the pediatric population for a wide variety of reasons. Chronic nonbacterial osteomyelitis, lymphoma and metastasis are amongst the most common indications. There is no consensus on a standardized imaging protocol, and due to insufficient reporting on technical data, a reproduction of the protocol or images from the published literature is hardly possible. Furthermore, studies addressing the precision, accuracy and clinical validity of whole-body MRI in children are lacking and, to date, there is no documented effect on morbidity and/or mortality. The results from our study could potentially be used to boost attempts toward standardization of technique, reporting and guidelines development.

## Electronic supplementary material

ESM 1 (PDF 74 kb)
