# Supplementary material for: Whole-body magnetic resonance imaging in children – how and why? A systematic review
Source: Pediatr Radiol. 2020 Jun 25;51(1):14–24. doi: 10.1007/s00247-020-04735-9 (PMC7796873; doi:10.1007/s00247-020-04735-9)
Supplement: Supplementary file 1 — (PDF 74 kb) [file 247_2020_4735_MOESM1_ESM.pdf]

Supplementary 1

Ovid MEDLINE(R) and Epub Ahead of Print, In-Process & Other Non-Indexed Citations and Daily <1946 to November 02, 2018> Search date 5. Nov. 2018

1 Whole Body Imaging/ (4371)

2 ((whole body or wholebody or whole-body or full body or entire body or complete body or total body or full-length or uncut) adj3 (magnetic resonance imaging or MRI or MR imaging or MR-imaging or NMR or tomography MR or MR tomography)).ti,ab,kw. (1818)

3 Magnetic Resonance Imaging/ (362220)

4 (whole body or wholebody or whole-body or full body or entire body or complete body or total body or full-length or uncut).ti,ab,kw. (151508)

5 1 or 4 (153445)

6 3 and 5 (3815)

7 2 or 6 (4509)

8 adolescent/ or exp child/ or exp infant/ (3339152)

9 (child* or adolescen* or teen* or kids or youth* or preschool or infant* or newborn or paediatric* or pediatric*).ti,ab,kw. (1926848)

10 8 or 9 (3836142)

11 7 and 10 (786)

Embase (OVID) <1974 to 2018 November 02>. Search date 5. Nov. 2018

1 whole body mri/ (2247)

2 ((whole body or wholebody or whole-body or full body or entire body or complete body or total body or full-length or uncut) adj3 (magnetic resonance imaging or MRI or MR imaging or MR-imaging or NMR or tomography MR or MR tomography)).ti,ab,kw. (3079)

3 1 or 2 (3922)

4 juvenile/ or adolescent/ or child/ (2307420)

5 (child* or adolescen* or teen* or kids or youth* or preschool or infant* or newborn or paediatric* or pediatric*).ti,ab,kw. (2293593)

6 4 or 5 (3354785)

7 3 and 6 (776)

Cochrane (Wiley), Search date 5.nov.2018

#1 - MeSH descriptor: [Whole Body Imaging] explode all trees MeSH - 65

#2 - ("whole body" or wholebody or whole-body or "full body" or "entire body" or "complete body" or "total body" or full-length or uncut):ti,ab,kw - (Word variations have been searched) S - 8829

#3 - MeSH descriptor: [Magnetic Resonance Imaging] explode all trees MeSH - 7418

#4 - (#1 or #2) and #3 - 119

#5 - (("whole body" or wholebody or whole-body or "full body" or "entire body" or "complete body" or "total body" or full-length or uncut) NEAR/3 ("magnetic resonance imaging" or MRI or "MR imaging" or MR-imaging or NMR or "tomography MR" or "MR tomography")):ti,ab,kw - (Word variations have been searched) S - 141

#6 - #4 or #5 - 221

#7 - MeSH descriptor: [Infant] explode all trees MeSH - 15055

#8 - MeSH descriptor: [Child] explode all trees - MeSH - 1422

#9 - MeSH descriptor: [Adolescent] explode all trees MeSH - 97811

#10 - (child* OR adolescen* OR teen* OR kids OR youth* OR preschool OR infant* OR newborn OR paediatric* OR pediatric*):ti,ab,kw - (Word variations have been searched) S - 224945

#11 - #7 or #8 or #9 or #10 - 224945

#12 - #6 and #11 - 45
